# Inhibitor of differentiation 4 (Id4) is a potential tumor suppressor in prostate cancer

**DOI:** 10.1186/1471-2407-9-173

**Published:** 2009-06-07

**Authors:** Jason PW Carey, Ananthi J Asirvatham, Oliver Galm, Tandeih A Ghogomu, Jaideep Chaudhary

**Affiliations:** 1 Department of Biology, Center for Cancer Research and Therapeutics Development, Clark Atlanta University, Atlanta, GA 30314, USA; 2 Medizinische Klinik IV, Universitaetsklinikum Aachen, RWTH Aachen, Pauwelsstrasse 30, 52074 Aachen, Germany

## Abstract

**Background:**

Inhibitor of differentiation 4 (Id4), a member of the Id gene family is also a dominant negative regulator of basic helix loop helix (bHLH) transcription factors. Some of the functions of Id4 appear to be unique as compared to its other family members Id1, Id2 and Id3. Loss of Id4 gene expression in many cancers in association with promoter hypermethylation has led to the proposal that Id4 may act as a tumor suppressor. In this study we provide functional evidence that Id4 indeed acts as a tumor suppressor and is part of a cancer associated epigenetic re-programming.

**Methods:**

Data mining was used to demonstrate Id4 expression in prostate cancer. Methylation specific polymerase chain reaction (MSP) analysis was performed to understand molecular mechanisms associated with Id4 expression in prostate cancer cell lines. The effect of ectopic Id4 expression in DU145 cells was determined by cell cycle analysis (3H thymidine incorporation and FACS), expression of androgen receptor, p53 and cyclin dependent kinase inhibitors p27 and p21 by a combination of RT-PCR, real time-PCR, western blot and immuno-cytochemical analysis.

**Results:**

Id4 expression was down-regulated in prostate cancer. Id4 expression was also down-regulated in prostate cancer line DU145 due to promoter hyper-methylation. Ectopic Id4 expression in DU145 prostate cancer cell line led to increased apoptosis and decreased cell proliferation due in part by an S-phase arrest. In addition to S-phase arrest, ectopic Id4 expression in PC3 cells also resulted in prolonged G2/M phase. At the molecular level these changes were associated with increased androgen receptor (AR), p21, p27 and p53 expression in DU145 cells.

**Conclusion:**

The results suggest that Id4 acts directly as a tumor suppressor by influencing a hierarchy of cellular processes at multiple levels that leads to a decreased cell proliferation and change in morphology that is possibly mediated through induction of previously silenced tumor suppressors.

## Background

The Id genes (Id1, Id2, Id3 and Id4) are part of the broader basic helix loop helix family. The basic helix-loop-helix (bHLH) proteins are DNA binding proteins that regulate tissue-specific transcription within multiple cell lineages [1]. Hetero- or homo-dimerization-dependent DNA binding activity of class A bHLH proteins are regulated to a large part by the class D HLH inhibitors of differentiation (Id) gene family [2]. The Id proteins lack the DNA binding basic domain but have intact HLH domain [2,3]. This domain configuration allows the Id family to dimerize with bHLH transcription factors, but the lack of the basic domain renders the Id-bHLH dimer transcriptionally inactive, as it fails to bind and regulate promoter activity of genes dependent on E-box (CANNTG) response element [4]

The four different isoforms of Ids (Id1, Id2, Id3 and Id4) have a highly conserved HLH domain but divergent N- and C-terminal domains. This sequence divergence may account for protein-specific interactions possibly resulting in differential functions of Id proteins [5-7]. Although all Id proteins interact with E-proteins, but isoform specific bHLH and non-bHLH interactions are known to occur. For example, interaction of a) Id2 directly with hypophosphorylated pRb protein family [8,9] and polycystins [10] b) Id2 and Id4 with OLIG (class A bHLH, [11]) c) Id1 and calcium/calmodulin-dependent serine protein kinase (CASK) [12] and d) Id1 and Id3 with *v-ets *erythroblastosis virus E26 oncogene homolog (Ets) [13] and Paired box transcription factor (Pax) homeodomain containing proteins [14]. Consistent with gene specific interactions, the Id proteins also exhibit isoform specific functions such as modulation of breast cancer 1, early onset (BRCA1) promoter activity by Id4 [15,16], localization of Id1 to the centrosomes [17] leading to accumulation of cells with abnormal centrosome number and induction of apoptosis by Id2 in myeloid precursors, osteosarcoma [18] and neuronal cells [19] by an HLH independent mechanism.

In general, Id proteins (Id1-3) promote cell proliferation [20-22]. Consequently, the expression of Id proteins is generally high in proliferating cells that is down-regulated as a prerequisite for exit from the cell cycle during differentiation [23]. Consistent with this observation, an increased expression of various Id isoforms has been detected in many cancers [24-32].

In comparison to Id1, Id2 and Id3, the function of Id4 is less understood and often conflicting. Both tumor promoting and tumor suppressor roles of Id4 have been reported in many cancers. Tumor suppressor roles of Id4, based on its loss of expression in association with promoter hypermethylation have been suggested in leukemia [33], breast [34,35], colorectal [36] and gastric cancers [37]. The pro-tumor effect of Id4 is observed in bladder [38] and rat mammary gland carcinomas [39]. Id4 is also the only Id gene that is deregulated by a t(6;14)(p22;q32) chromosomal translocation in a B-cell acute lymphoblastic leukemia [40] and B-cell precursor acute lymphoblastic leukemia (BCP-ALL) [41].

The expression of Id4 in prostate epithelial cells is particularly interesting. Id4 appears to be androgen regulated in normal prostate epithelial cells [42] and in the androgen sensitive prostate cancer cell line LNCaP [43]. Id4 expression is undetectable or weakly expressed in androgen independent DU145 cells whereas its expression is observed (low) in PC3 prostate cancer cell lines [43]. LNCaP prostate cancer cells are generally considered less tumorigenic and more differentiated as compared to highly tumorigenic DU145 and PC3 prostate cancer cells. These observations suggest that Id4 expression may be associated with the state of differentiation and tumorigenic potential of prostate epithelial cells. This hypothesis was examined in the present study by over-expressing Id4 in androgen receptor negative DU145 and PC3 prostate cancer cells. Our results, demonstrate that Id4 attenuates cell cycle by promoting an S-phase arrest and induces androgen receptor expression. Id4 expression is also significantly reduced in prostate cancer samples as determined through data mining.

## Methods

### Cell Lines and Cell Culture

Human prostate cancer cell lines DU145 and LNCaP were obtained from American Type Culture Collection (ATCC, Rockville, MD). The DU145 cells were cultured in F12-BCS-A (Ham's F12 (Gibco, Carlsbad, CA) medium containing 10% Bovine Calf Serum (Hyclone) with appropriate antibiotics (pen/strep, fungizone, and gentamycin). LNCaP cells were cultured in RPMI-10% Fetal Calf Serum (FCS) and antibiotics. The normal human prostate epithelial cells (PrEC) were obtained from Cambrex (Baltimore, MD) and were cultured in PrEGM (Cambrex, Baltimore, MD) for approximately 10–15 doublings. Cells were cultured at 37°C in a fully humidified atmosphere containing 5% CO_2_.

### 5'-Aza-2-Deoxycytidine (5-AZA-CdR) Treatment

The DU145 cells were cultured in F12-BCS-A containing 4 uM of 5-AZA-Cdr (4 uM). The media with freshly added 5-AZA-Cdr was changed every 24 hours for 96 hours before harvesting the cells for RNA. The gene expression was analyzed before and after 5-AZA-Cdr treatment on the reverse transcribed RNA using gene specific primers.

### Plasmids and transfections

The full length human Id4 cloned into pCMV vector (pCMV-Id4) was a generous gift from Dr. Mark Israel. The pCMV-Id4 vector and pCMV vector alone was transfected in sub-confluent (60%) DU145 and PC3 cells grown in six well plates using TransIT-prostate transfection reagent cocktail (10 μl TransIT-prostate reagent, 5 μl prostate boost reagent (Mirus Bio) and 2 μg pCMV-Id4 DNA in 200 ul of serum free media). The culture media was changed once after an overnight incubation with the cocktail. Forty-eight hours after transfection, the cells with incorporated pCMV-Id4 were selected by incubation in fresh media containing 350 μg/ml G418 (Invitrogen) for one week with media change every 2 days. Following this selection cycle, the transfected cells were passaged once in F12-BCS-A and then re-exposed to F12-BCS-A with 350 μg of G418/ml for an additional week (second G418 selection). This approach ensured the survival of only transfected cells. Simultaneous experiments were also performed in which cells were transfected with no DNA (control, parental) or with pCMV DNA (transfection control). The G418 selection procedure described above resulted in no surviving DU145 parental cells. The cells were grown to confluence (80%), trypsinized (0.25% v/v trypsin and 0.03% w/v EDTA in calcium- and magnesium-free phosphate buffered saline), counted, and plated at a 1:2 dilution in new 100-mm plates.

### RNA preparation

Total RNA was extracted using TRIzol (Invitrogen, Carlsbad, CA) as described previously [43]. The final RNA pellet was re-suspended in diethylpyrocarbonate (DEPC)-treated H_2_O at a concentration of 1 mg/ml and stored at -80°C until analysis.

### Proliferation assay

DU145 cells were cultured at sub-confluent densities (growth permissive, 40%) in 24 well plates (40% confluency) and serum starved for 48 hours. The cells were then treated with 10% BCS for 20 h followed by a 6 h incubation with [3H] thymidine. Counts per min (cpm) of [3H] thymidine incorporated into DNA were determined and normalized to the total DNA per well. Total DNA content was determined by SYBR green fluorescent assay [44].

### Gene Expression

RNA (2 μg) was reverse transcribed in a final volume of 25 μl as per standard protocols (RT-Mix: 20 U RNAout (Invitrogen, Carlsbad, CA); 1.25 mM each of dNTP's; 250 ng oligo dT (Promega, Madison, WI), 10 mM dithiothreitol, and 200 U MMLV reverse transcriptase (Invitrogen) in the MMLV first-strand synthesis buffer (Invitrogen)). The RNA was denatured for 10 min at 65°C, and then cooled on ice before addition of RT mix and enzyme. The reverse transcriptase reaction was carried out at 42°C for 1 h.

Each PCR reaction was performed with 250 pg reverse-transcribed DNA using published protocols [43]. The possible contamination of RNA with DNA was distinguished by performing the RT reaction without MMLV reverse transcriptase enzyme (-MMLV RT). The PCR-based amplification reactions were carried out in duplicate on each reverse-transcribed RNA sample using gene specific primer sequences as indicated in respective figure legends. Simultaneous PCR reactions were also carried out using primers designed to β-actin to monitor the qualitative and quantitative efficiency of the RT-PCR reactions. The identity of each of the corresponding PCR products was size and sequence/restriction digest confirmed.

### Real-Time PCR

Reverse transcribed RNA from DU145, DU145-Id4 and DU145-pCMV cells were used for real time quantitative gene expression analysis based on the TaqMan chemistry. The validated probes for Id4, β-actin (control), E-cadherin (CDH1), p21 (CDKN1A) and p53 were obtained from Applied Biosystems. Amplification of target sequences was detected with ABI7900HT sequence detection system (Applied Biosystems, Foster City, CA) and analyzed with ABI prism software (Applied Biosystems). All PCR reactions were performed in a final volume of 50 μl according to the manufacturer's instructions (Applied Biosystems). The cycle threshold (Ct) was used to calculate relative amounts of target RNA.

### Immuno cytochemistry

The cells lines were cultured (60% confluency) in 8 chamber slides (Lab-Tek) at 37°C as described above. After 2 days in culture, the media was removed and the adherent cells were washed once with PBS. The cells were then fixed in cold methanol (20C) for 1 hour and stored at -20C. The fixed adherent cells on slides were equilibrated at 4C before further processing. The slides were incubated in 3% Bovine Serum Albumin (BSA) in PBS for 1 hour at 4C in order to block non-specific sites. The slides were then incubated with anti-Androgen Receptor-antibody (1:10,000, PG21, Millipore) or anti-actin antibody (1:10,000, H-196, Santa Cruz) for 1 hour at 4C. After primary antibody reaction, the cells were washed 3 times in PBS for 5 minutes each and then incubated with Alexa Fluor 594 anti-rabbit IgG (1:10, Invitrogen) at 4C for 1 hour. The slides were then rinsed for 5 × 5 minutes in PBS, counter stained with DAPI (VWR), post fixed with 3.7% formalin in PBS for 15 minutes and mounted with Glycerol. Cells were examined using a Fluorescent Microscope (Zeiss) and images captured via AxiomCam/AxioVision (v 4.4).

### Western Blot Analysis

The prostate cancer cell lines were cultured on 150 mm plates in their respective media. Cells (5 × 10^6^) were dissociated off the plate by adding ice cold PBS and scrapping with a cell scraper. Total cellular protein was prepared as described previously [43].

20 ug of total protein was separated by 4–20% SDS-polyacrylamide gel (BioRad) and subsequently blotted onto a nitrocellulose membrane (BioRad). The blotted nitrocellulose membrane was subjected to western blot analysis using protein specific antibodies (Id4: Aviva ARP32335_T100) as mentioned above. After washing with 1× PBS with 0.5% Tween 20, the membranes were incubated with a secondary antibody against anti-rabbit IgG (Pierce Rockford, IL) and the signal was visualized using the Super Signal West Dura Extended Duration Substrate (Pierce).

### Flow Cytometric Analysis of Cell Cycle Progression and apoptosis

The DU145 and PC3 cells were cultured in 24 well plates to a sub confluent density. After the culture the cells were collected by trypsinization and washed with phosphate buffered saline. The cells were then fixed with 70% ethanol and stored at -20°C overnight. The following day, cells were washed twice with ice cold phosphate buffered saline PBS/FCS (10%) followed by a final wash in 1× PBS. The cells were then finally resuspended 1 ml of PBS (1×) containing 50 μg/ml RNase A, 0.1% TritonX-100 and 1 mM EDTA and then incubated at 37°C for 30 minutes. Finally, 20 μg/ml of propidium iodide was added. Data acquisition and analysis were performed on a BD FACScan flow cytometer (Dept. of Biology, Spellman College, Atlanta, GA). The cell cycle profiles were then analyzed using BD Cellquest Pro (for apoptosis, Sub G0) and MODFIT software cell cycle analysis. At least 10,000 cells in each sample were analyzed to obtain a measurable signal. All measurements were performed using the same instrument settings.

The fraction of cells in Sub G0 phase was used to detect apoptotic cells. An increase in Sub G0 cells is due to loss of fragmented DNA as a result of apoptosis from permeabilised cells (ethanol fixed) due to DNA fragmentation. The cells stained with a DNA intercalating dye like propidium iodide, results in a DNA profile representing cells in G1, S-phase and G2M phase while the apoptotic cells are represented by a sub G0/G1 population seen to the left of the G0/G1 peak [45-47].

### Statistical analysis

The ΔΔCt method (Applied Biosystems User Bulletin2; ABI PRISM 7700 detection system) was used for relative quantification of gene expression. The Ct values of the target genes from triplicate PCR reactions were normalized to the levels of β-actin (endogenous control) from the same cDNA preparations. The average Ct for each gene was calculated by subtracting the Ct of the sample RNA from that of the control RNA. This value or ΔCt reflected the relative expression of the treated sample compared with the control and became the exponent in the calculation for amplification 2Δ^Ctcont-ΔCtsample^, the equivalent to the fold change in expression. A statistically significant difference between various treatments and/or cell lines was determined by student's t-test.

### DNA Methylation Analysis

The methylation status of the Id4 promoter region was analyzed using methylation-specific PCR (MSP) [48]. This assay entails the initial modification of genomic DNA by sodium bisulfite, converting all un-methylated cytosines to uracils, but leaving the methylated cytosines unchanged. Subsequently, the DNA region of interest was amplified in two separate reactions with primer pairs specific for either the methylated or the un-methylated sequence.

Genomic DNA was isolated using DNeasy kit (Qiagen). Approximately 1 μg of DNA was sodium bisulfite-modified and subjected to MSP as described previously [48]. MSP primers that specifically recognized un-methylated Id4 sequence were 5'-GGT AGT TGG ATT TTT TGT TTT TTA GTA TT-3' (sense) and 5'-AAC TAT ATT TAT AAA ACC ATA CAC CCC A-3' (antisense); primers specific for the methylated Id4 sequence were 5'-TAG TCG GAT TTT TCG TTT TTT AGT ATC-3' (sense) and 5'-CTA TAT TTA TAA AAC CGT ACG CCC CG-3' (antisense). The primers for the U reaction cover the bases -194 until -166 and -60 until -33, and the primers for the M reaction cover the bases -192 until -166 and -60 until -35 (relative to the transcription start site). Reactions were hot-started at 95°C for 5 min and held at 80°C before addition of 0.625 U of Taq polymerase (Sigma, St. Louis, MO). Temperature conditions for PCR were as follows: 35 cycles of 95°C for 30 sec, 58°C for 30 sec and 72°C for 30 sec, followed by 1 cycle of 72°C for 5 min. Normal DNA from peripheral blood was treated in vitro with *Sss*I methyltransferase (New England Biolabs, Beverly, MA) in order to generate a positive control for methylated alleles of Id4 [49]. PCR products were separated on 2.5% agarose gels and visualized by ethidium bromide staining.

## Results

### Id4 expression is down-regulated in prostate cancer

**Figure 1:**
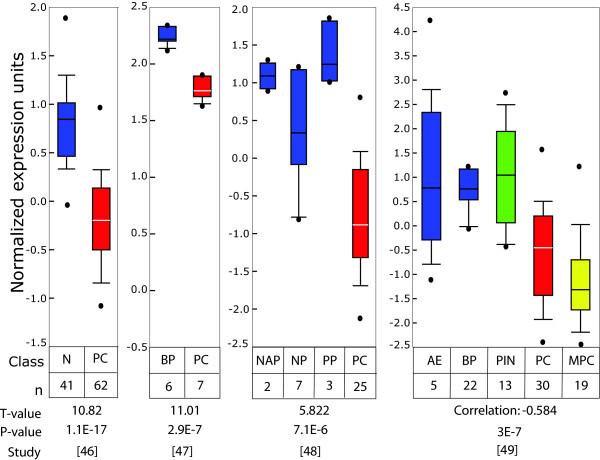
**Id4 expression in prostate cancer as determined from publically deposited microarray databases**. The Oncomine database was queried against Id4 and all prostate databases were analyzed. The box and whisker plots from four of these representative studies is shown [51-54]. Consolidated data from all these studies demonstrated that Id4 is significantly (T-values > 5 and low P-values < E-6) down-regulated in prostate cancer samples. Abbreviations: n: No. of samples in each analysis, PC: Prostate Cancer (Red), MPC: Metastatic prostate cancer (Yellow), NAP: Normal adjacent prostate (Blue), NP: Normal Prostate (Blue), PP: Post-pubertal prostate (Blue), BP: Benign prostatic hyperplasia (Blue), PIN: prostate intra-epithelial neoplasia (green).

Data mining of published microarray databases was used to determine the relative expression levels of Id4 in clinically relevant cases of prostate cancer as compared to controls and benign prostate hyperplasia. The consolidated Oncomine database [50] was queried against Id4 and all prostate databases were analyzed. Data from four of these representative studies is shown in Fig. 1. Between these four independent and unrelated studies [51-54], a total of 125 prostate cancer (PC), 13 metastatic prostate cancer (MPC), 53 normal controls (41 Normal (N), 2 normal adjacent prostate (NAP), 7 normal adult prostate (NP), 3 post pubertal prostate (PP)), 28 benign prostate hyperplasia (BP) and 13 prostate intra-epithelial neoplasia (PIN) were analyzed. Normalized Id4 expression analysis in these samples indicated: 1) High Id4 in benign prostate hyperplasia as compared to prostate cancer suggesting that decreased Id4 is a cancer specific event 2) High normal adjacent (NAP) and post pubertal (PP) Id4 gene expression that may signify the role of Id4 in maintaining normal prostate function and 3) Low Id4 expression in prostate cancer (metastatic prostate cancer < prostate cancer). Consolidated data from all these studies demonstrated that Id4 is significantly (T-values > 5 and low P-values < E-6) down-regulated in prostate cancer samples.

The molecular basis of Id4 down-regulation and its significance in prostate cancer was studied in a well characterized prostate cancer cell line DU145. The DU145 prostate cancer cell line is negative or expresses very low Id4 and hence is an excellent model to study mechanisms involved in its down-regulation. In contrast, the normal human prostate epithelial cells (PrEC) and prostate cancer cell lines (LNCaP) [43] express Id4.

### Id4 promoter methylation in DU145 cells

**Figure 2:**
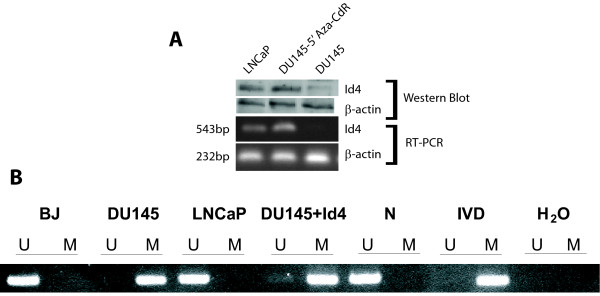
**(A) Id4 expression is generally absent in DU145 cells but present in androgen receptor positive LNCaP cells**. Treatment of DU145 cells with global de-methylation agent 5'-Aza-2-Deoxycytidine (5-AZA-CdR) leads to Id4 expression as determined by western blot analysis (upper panel) and reverse transcriptase polymerase chain reaction (RT-PCR, lower panel). Beta-actin was used as a loading and RT-PCR control. The primer pairs used for amplification were described in [43]. **(B) **Analysis of Id4 methylation in human fibroblasts (BJ) and the prostate cancer cell lines DU145, DU145-Id4 (DU145 cells with constitutively expressed Id4) and LNCaP by methylation specific PCR (MSP). The presence of a PCR band in lanes marked "M" indicates a methylated gene sequence, the presence of a PCR band in lanes marked "U" indicates an un-methylated gene sequence. Normal peripheral blood cells (N), *in vitro *methylated DNA (IVD) and water served as controls.

Results shown in Fig. 2A demonstrate that DU145 cells lack Id4 expression at the transcript and protein levels [43]. Lack of Id4 in DU145 cells is possibly due to its promoter hyper-methylation since treatment with global demethylation agent 5'-Aza-2-Deoxycytidine (5-AZA-CdR) resulted in the induction of Id4 gene and protein expression (Fig. 2A). The methylation at the CpG island located around the transcription start site leading to the epigenetic silencing of Id4 in several human solid tumors [33,34,36,37] have been reported. These results prompted us to investigate the methylation status of the Id4 promoter region in DU145 cells by MSP (Methylation Specific PCR). As shown in Fig. 2B, the Id4 promoter region in DU145 cells is also methylated as compared to Id4 expressing LNCaP cells, human fibroblasts and normal peripheral blood mononuclear cells. Thus, aberrant methylation of the promoter region appears to be associated with Id4 gene silencing in AR negative DU145 cells.

### Effect of Id4 on Cell Proliferation, Morphology and Apoptosis

Two weeks post-transfection, the expression of Id4 in DU145 cells transfected with pCMV-Id4 was evaluated with real time PCR analysis. A more than 10 fold increase in Id4 expression in DU145-Id4 cell line as compared to the parental DU145 cells confirmed that Id4 was being expressed in the pCMV-Id4 transfected cells (after 1^st ^G418 selection). Similar levels of Id4 (range 7.5–11 fold) was also observed after 2nd G418 selection. The Id4 expression in CMV transfected cells was negligible and was comparable to that in parental DU145 cells. Following this analysis, the cells were allowed to grow for at least 15 passages before subjecting to cellular/molecular analysis.

**Figure 3:**
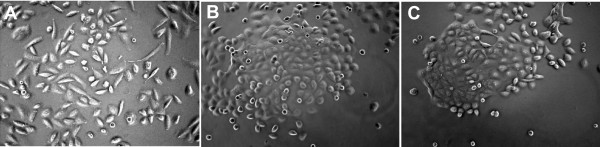
**Morphology (×100) of the DU145 and DU145-Id4 cell lines in culture**. **(A) **Parental DU145 cells show a more mesenchymal morphology that is long and spindle shaped. **(B & C) **The DU145-Id4 cells appear more like epithelial cells that tend to adhere to each other hence appear as clusters. The DU145-Id4 cell line shown above is at passage 28.

A change in morphology in DU145-Id4 cell lines was observed (Fig. 3). A significant observation was the appearance of "epithelial like" morphology that was associated with increased cell-cell adhesion of DU145-Id4 cells as compared to a mesenchymal morphology of the parental DU145 cells (Fig. 3).

**Figure 4:**
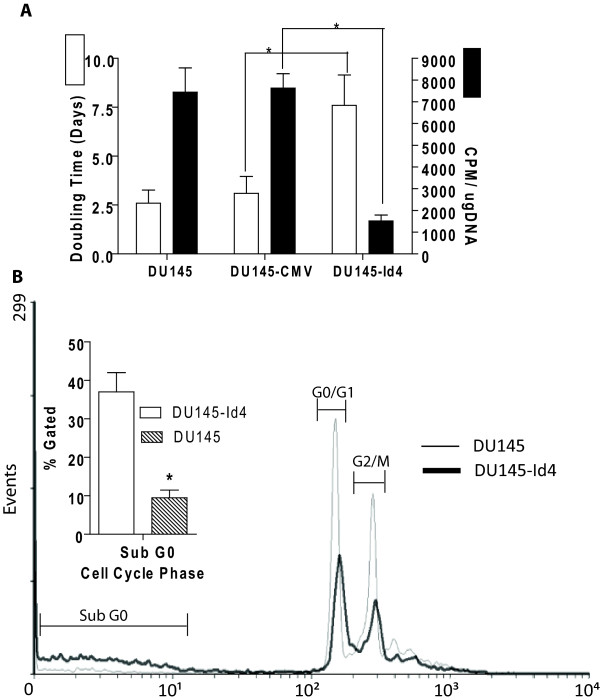
**(A): Doubling time (in days, White bars) and rate of proliferation (Black bars) of DU145, DU145-CMV and DU145-Id4 cells line in culture**. All the cell lines have matched passages. The DU145 cells were either transfected with no plasmid (DU145), CMV vector alone (DU145-CMV/EV) or pCMV-Id4 (DU145-Id4). White Bars: The average doubling time of DU145-Id4 cells as compared to DU145 and DU145-CMV. The data is an average cell count from four passages. The doubling time was calculated by counting days required for the number of plated cells (usually at 40% confluence) to reach confluence (80%). For DU145-Id4 cells, this average is from passage 13–16. A highly significant increase in the doubling time (approximately 2.5 fold) (P < 0.001) suggests that Id4 over-expression leads to a decrease in proliferation. Black Bars: 3H thymidine incorporation assay. 3H thymidine incorporation (CPM) was evaluated as a measure of rate of proliferation. The counts per minute (CPM) of incorporated 3H-thymidine was normalized to total DNA. The data represents CPM/ug DNA of cell lines at passages 14 and 15. The data is represented as mean ± SEM of three experiments performed in triplicates. *: P < 0.001. **(B) **FACS analysis of cell cycle parameters in DU145 and DU145-Id4 cell lines. The cell cycle profile showing fraction of cells in each phase of the cell cycle in DU145-Id4 and DU145 cells is shown. The data is representative of at least three experiments. The inset shows the fraction of cells in sub-G0 phase (normalized to 10,000 cells). The cells in sub-G0 phase have low DNA content possibly due to apoptosis. The mean ± SEM of three experiments performed in triplicate is shown (*: P < 0.001).

The slow but continuous rate of proliferation suggested that DU145-Id4 cells have not undergone senescence (at least until passage 28). In vitro growth curves (Doubling time, Fig. 4A) indicated a decrease in cell proliferation (increased doubling time) in DU145-Id4 cells as compared to non-transfected DU145 and DU145-pCMV (empty vector transfected) cells. The decreased proliferation of DU145-Id4 cells as compared to DU145 and DU145-CMV were also reflected in 3H-thymidine incorporation assay that measures DNA synthesis (Fig. 4A). Fluorescent Assisted Cell Sorting (FACS) was then used to understand the effect of Id4 on cell cycle. Two different approaches were used: The first approach was used to determine the fraction of cells in the Sub-G0 population as an estimate of apoptosis and the second approach, using ModFit software was used to determine the fraction of cells in each phase of the cell cycle. The DU145-Id4 cells demonstrated increased apoptosis as indicated by increased cell counts in sub-G0 phase as compared to DU145 cells (Fig. 4B).

**Figure 5:**
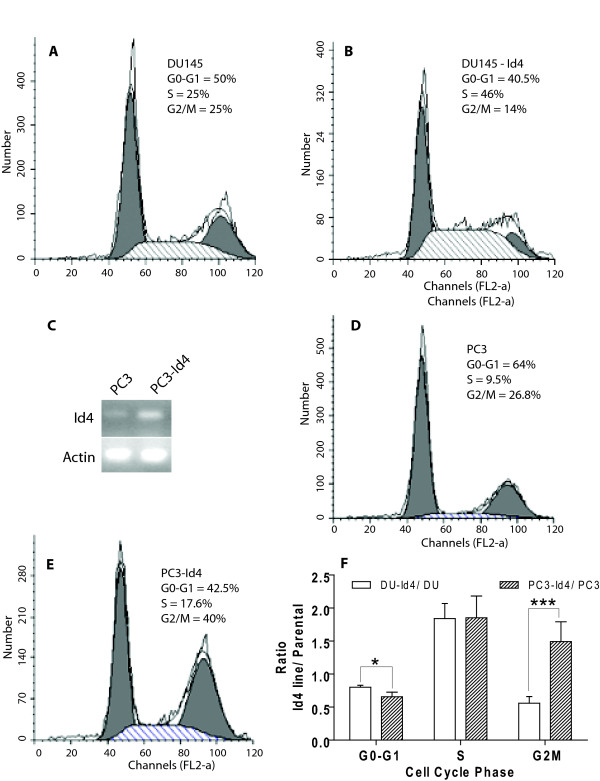
**FACS analysis of cell cycle parameters in DU145, DU145-Id4, PC3 and PC3-Id4 cell lines**. The cells were serum starved for 24 hours in order to synchronize the cell cycle. The cells were then serum stimulated (10% BCS) in order to determine the effect of Id4 on cell cycle. **A and D**: Serum treated DU145 and PC3 cells respectively, **B and E**: Serum treated DU145-Id4 and PC3-Id4 cells respectively. The cell cycle analysis and the fraction of cells in each phase (G0-G1, S and G2/M, indicated as % cells) were determined using ModFit cell cycle analysis software. The data is representative of triplicate experiments. Of note is the number of cells (scale) represented on the Y-axis. **C**: Reverse Transcriptase polymerase chain reaction demonstrating the expression of Id4 in PC3 (lane 1) and PC3 cells stably transfected with Id4 expression plasmid (Lane 2). Weak Id4 expression was observed in PC3 cells whereas significantly higher expression was observed in PC3-Id4 cells (representative of 3 different PCR reactions). **F**: The ratio of cells in each phase. The ratio was calculated by dividing the % cells in Id4 expressing cells by parental cells in each phase of cell cycle. This calculation was used to normalize and compare the data between two cell lines. The error bars represent standard error of mean calculated from three different experiments (*: P > 0.05 and ***: P > 0.001).

The decreased proliferation of DU145-Id4 cells was due to lower number of cells in the G0/G1 and G2/M phase (Fig. 5B) as compared to DU145 cells (Fig. 5A). A significant observation was the increase in the number of cells in the S-phase in DU145-Id4 cells (Fig. 5B) as compared to DU145 cells (Fig. 5A). Collectively, these results demonstrated that Id4 expression induces a change in cell morphology/adhesion, decreased proliferation possibly to due to an S-phase arrest and increased apoptosis.

The effect of Id4 expression on PC3 was also investigated to confirm the results obtained from DU145 cells. PC3 cells are weakly positive for Id4 expression (Fig. 5C) but nevertheless provide a good model to investigate the effect of ectopic Id4 expression on prostate cancer cells (Fig. 5C). Although detailed analysis of PC3-Id4 cell line is ongoing but initial results on cell cycle profiling by FACS analysis demonstrated striking similarities to the DU145-Id4 cells line. The PC3-Id4 cell line also demonstrated an S-phase arrest (Fig. 5E) as compared to PC3 cells (Fig. 5D). The cell cycle profile between PC3 cells and PC3 cells transfected with empty vector were similar (data not shown). The ratio of cells in S-phase (% Cells in Id4 cell lines/% cells in parental lines in each corresponding cell cycle phase) in DU145-Id4 and PC3-Id4 were strikingly similar (Fig. 5F). The data normalization also suggested that unlike DU145-Id4, the PC3-Id4 cell line may have an additional block at G2/M phase.

### Gene Expression Changes in DU145-Id4 cell lines

**Figure 6:**
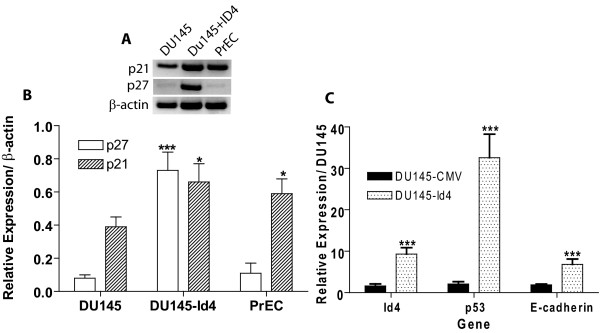
**(A): RT-PCR and semi-quantitative expression levels of cyclin dependent kinase inhibitors p27 and p21 in DU145, DU145-Id4 and PrEC (normal prostate epithelial) cells**. **(B) **Semi-quantitative analysis of RT-PCR results shown in (A). The intensity of each p27 and p21 band was normalized to constitutively expressed beta actin. The RT-PCR data and semi-quantitative analysis (expressed as mean ± SEM) is representative of 3 different RT and corresponding PCR reactions (***: P < 0.001, *: P < 0.05 as DU145). The following primers were used: p27 (CDKN1A): Forward 5'-TCA AAC GTG CGA GTG TCT AA and Reverse 5'-ACG TTT GAC GTC TTC TGA GG, p21: Forward 5'-CGA CTG TGA TGC GCT AAT G and Reverse 5'-TTA GGG CTT CCT CTT GGA GA, β-actin: Forward-5' AGA AAA TCT GGC ACC ACA CC, Reverse-5' GGG GTG TTG AAG GTC TCA AA. **(C)**: Real time analysis of Id4, p53 and E-cadherin gene expression in DU145, DU145-CMV and DU145-Id4 cell lines. The data performed in triplicate is mean ± SEM from cells at passages 18, 20 and 23. The real time data is normalized to the constitutively expressed gene beta-actin. The relative expression levels were calculated by the Δ Ct method as described in materials and methods section. The RT-PCR data is representative of 3 different RT and corresponding PCR reactions (***: P < 0.001, as compared to DU145).

The likely candidates involved in suppressing proliferation are the cyclin dependent kinase inhibitors (CDKNI) p21 and p27. The expression of p21 and p27 in DU145-Id4 cells was determined by semi-quantitative RT-PCR analysis (Fig. 6A and 6B). The expression of p21 in DU145-Id4 and normal prostate epithelial cells (PrEC) was significantly higher as compared to parental DU145 cells (P < 0.05). The expression of p27 was also significantly higher in DU145-Id4 cells as compared to DU145 cells in which it expression was absent or below detection levels (P < 0.001). A highly significant increase (over 15 fold, Real Time PCR) in p53 transcript level was observed in DU145-Id4 cell line as compared to DU145 cells (Fig. 6C). Thus, the decrease in DU145-Id4 cell proliferation appears to be mediated in part by increased expression of classical tumor suppressors' p27 and p21.

At the molecular level, the transition towards "epithelial type" morphology and increased cell adhesion of DU145-Id4 cells could be due an increase in E-cadherin expression as determined by real-time PCR analysis (Fig. 6C). The E-cadherin expression in DU145-Id4 cells was at least five fold higher than DU145 cells (Fig. 6C).

**Figure 7:**
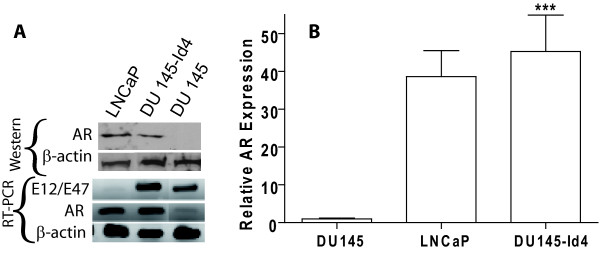
**(A): Western blot analysis of Androgen receptor expression analysis (Upper Panel)**. Lower Panel: Expression of androgen receptor (AR) and E2A (E12/E47) bHLH transcription factor by RT-PCR. The gain of androgen receptor expression in DU145-Id4 cells as compared to DU145 cells at the transcript and protein level is evident. The total RNA and protein was purified from DU145-Id4 at passage 28 and analyzed for the expression studies. As controls, the parental, mock transfected DU145 cells (AR -ve) and LNCaP (AR +ve) cell lines were used. The expression of beta-actin was used as loading and RT-PCR control. The data is representative of at least three different RT-PCR reactions and Western blot analysis. The following primers were used: E2A F-5' CAC CAG CCC TCA TGC ACA ACC, R-5' CTC CAA CCA CAC CTG ACA C and androgen receptor (AR): F-5' ATG GTG AGC AGA GTG CCC TA and R-5' GTG GTG CTG GAA GCC TCT CCT. **(B) **Real time PCR analysis, performed on the same batch of reverse transcribed RNA used in panel A confirms the RT-PCR data. The fold change in AR expression is normalized to beta-actin (3 different RT reactions) and calculated by the Δ Ct method as described in materials and methods section (*** P < 0.001).

**Figure 8:**
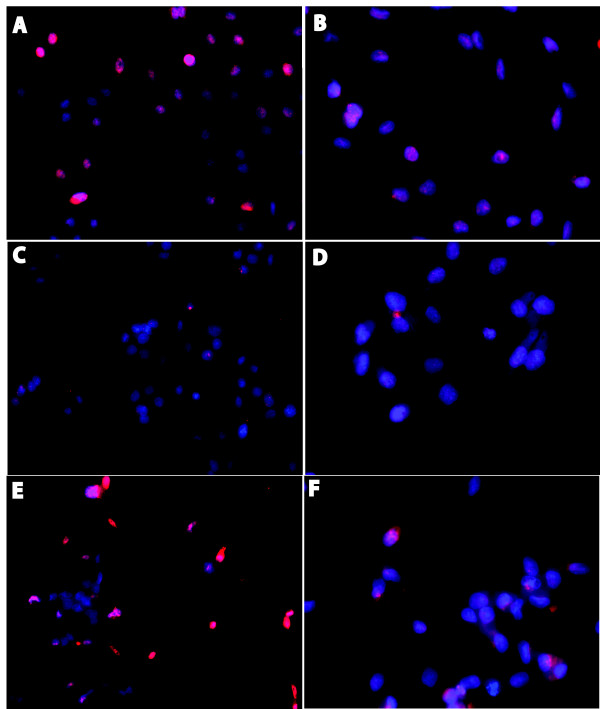
**Fluorescence immuno-cytochemistry demonstrating expression and localization of androgen receptor (AR) at the cellular level**. The cells were processed for immuno-cytochemistry as described in the materials and methods section. The red fluorescence demonstrates AR (panels A, C, E) or β-actin (panels B, D, F). The cells were counterstained with DAPI (blue) to reveal the nucleus. The red (antigen specific) and blue (nucleus) images were superimposed to demonstrate cellular localization of the antigen (AR or β-actin). Androgen receptor expression was localized primarily to the nucleus in LNCaP (panel E, AR+ve control) and DU145-Id4 (Panel A). In contrast, β-actin expression was primarily cytoplasmic (panels B, D, F). The AR expression in parental DU145 cells was not observed (panel C). Panels A and B: DU145-Id4, Panels C and D: DU145 and Panels E and F: LNCaP. The anti-AR and anti-β actin antibodies were highly specific and displayed no non-specific binding as determined by western blot analysis (e.g. Fig 7A). The photomicrographs (×200) are representative of at least 3 different experiments performed in replicates.

One of the hallmarks of the DU145 cell line, a representative of androgen depletion-independent cancers, is the loss of androgen receptor. Based on our observations that ectopic expression of Id4 can decrease proliferation, increase apoptosis and induce a transition towards an epithelial phenotype, we considered the possibility that Id4 may re-induce AR expression. Indeed, Id4 over expression led to a highly significant increase in the expression of androgen receptor in DU145 cells as determined by western blot analysis (Fig. 7A, upper panel), RT-PCR (Fig. 7A, lower panel) and real time PCR (Fig. 7B). The androgen receptor expression in DU145-Id4 cells was further confirmed at the cellular level by immuno-cytochemistry (Fig. 8A). As expected, the AR expression in DU145-Id4 cells was localized primarily to the nucleus (Fig. 8A). A similar AR expression was also observed in AR +ve LNCaP cells (Fig 7A,B and Fig. 8E). Consistent with our RT-PCR and western blot analysis, AR expression was largely absent in parental DU145 cells (Fig 7A,B and Fig. 8C). Studies have demonstrated that DU145 cells are negative for androgen receptor due to promoter methylation [55] or express low levels of androgen receptor [56]. Therefore, induction of AR expression by Id4 can be due to a number of pathways, most notably through demethylation of AR promoter and/or induction of alternate transcriptional pathways required for AR expression.

The Id family of bHLH proteins interacts with and negatively regulates the bHLH proteins that generally induce differentiation and block proliferation. Primary among bHLH-Id interaction partners are the tumor suppressor E2A proteins E12/E47 [57]. At the transcriptional level, Id proteins also down regulate E2A [58,59]. Unexpectedly, Id4 expression was associated with increased expression of E12/E47 transcript (Fig. 7A). The induction of E12/E47 by Id4 therefore seems to be an isoform specific function [58,59]. Thus, one of the mechanism by which Id4 may exert its tumor suppressive effect is through up-regulation of E12/E47 bHLH proteins that may in turn up-regulate p21 [60] expression at transcriptional level.

Collectively, our results suggest that Id4 acts as a potential tumor suppressor, possibly through multiple pathways. The underlying molecular mechanisms are likely to be complex that may involve gene specific Id4 targets or more global, involving changes in response to trophic factors and modulation of overall transcriptional machinery.

## Discussion

In this report we demonstrate that Id4 acts as a putative tumor suppressor. The tumor suppressor role of Id4 appears to be unique as compared to other members of the Id gene family (Id1, Id2 and Id3) that may act as oncogenes or co-operating oncogenes in many cancers [24,27,30].

Hypermethylation of CpG islands near gene promoter regions is associated with transcriptional inactivation and represents an important mechanism of gene silencing in carcinogenesis [61,62]. Epigenetic silencing (promoter hyper-methylation) of Id4 in cancers from different organs such as in T-/natural killer acute lymphoblastic leukemia [33], gastric [37], breast [35] and colorectal cancers [36] suggests its role as a putative tumor suppressor. Analysis of public microarray databases provides strong evidence that Id4 is down regulated in prostate cancer also [51-54]. At the mechanistic level, the transcriptional inactivation of Id4 is associated with aberrant promoter methylation in a model prostate cancer cell line DU145.

At the molecular level, its core function as a dominant negative regulator of bHLH activity is conserved with its other three family members (Id1, Id2 and Id3). Id4 efficiently dimerizes with E proteins and blocks trans-activation of E box dependent genes [63]. Beyond this conserved role, the non-bHLH interaction partners that define global or cell type specific Id4 functions are largely unknown. For example, interactions of Id2 with Rb [8,9] and polycystins [10], Id1 and Id3 with Ets [13] transcription factors largely contribute to their oncogenic potential by releasing cell cycle blockade at multiple levels [32].

The effect of Id4 leading to decreased proliferation and S-phase arrest in DU145 prostate cancer cell line may be due to increase in the expression of tumor suppressors E-cadherin, p27, p21 and the bHLH transcription factors E12/E47 [64-67] and/or activation of previously silenced tumor suppressors. The increase in the transcript of p27 and p21 suggests that Id4 over-expression modifies intracellular transcriptional pathways leading to increased p27 and p21 expression itself. The increase in E-cadherin could be due to neutralization of bHLH transcriptional inhibitors [68] by Id4 or a secondary event elicited by increased p27 expression [69].

The p21 levels could be partially responsible for the increased p53 expression [70] in DU145-Id4 cells. However, the S-phase arrest and apoptosis appears to be p53 dispensable since p53 is expressed in a mutant form (p53 mt/mt) in DU145 cells [71]. A similar S-phase arrest observed in PC3-Id4 cells that are null for p53 further supports this mechanism. The p53 mutations in DU145 cells are within the DNA binding domain (P223L and V274F) that abrogates its transcriptional activity. The rate of apoptosis in DU145-Id4 expressing ectopic wild type p53 or alternate apoptotic pathways involving NFkB, Bcl-2, Bcl-xl needs to be investigated to further clarify mechanism of action of Id4 in apoptosis and S-phase arrest. The epigenetic silencing of p21 and p16 in PC3 cells as reported previously [72,73] but not in DU145 (this study) suggest that the molecular mechanisms involved in S-Phase and G2/M phase arrest in PC3 cells could be unique that remains to be investigated. Our data, however does confirm that Id4 is involved in blocking the cell cycle of prostate cancer cell lines DU145 and PC3. It is also speculated that the mechanism by which Id4 promotes S-phase arrest could be independent of CDKNIs and involve mechanisms that remains to be investigated.

Re-expression of functional AR can be achieved by treating the DU145 cells with non-selective de-methylating agents such as 5-aza-2' deoxycytidine [55,74] or treatment with growth factor NGF [75]. However, low AR expression in spite of its promoter methylation reportedly exists in DU145 cells [56]. An increased AR expression could therefore involve restoration of a transcriptional network by Id4 in DU145 cells at the non-epigenetic (signaling, transcriptional) level. A similar mechanism could also be operational for p53 expression that involve Id4 directly, or indirectly through increased p21 expression [70].

Evidence also suggests that Id4 can act as promoter of neoplastic transformation/growth. In breast cancer cells, Id4 and BRCA1 are in a negative feedback loop [15,16,76]. In rat mammary gland carcinoma, Id4 expression is associated with proliferation and invasiveness [39]. In other studies, Id4 was down regulated due to promoter hypermethylation in breast cancer [34,35]. Therefore, even in cancers arising from the same organ such as the breast, Id4 may have opposing effects [15,16,34,35,39,76]. Id4 is also over-expressed in acute lymphoblastic leukemia due to a t(6;14)(p22;q32 translocation [40]. Thus, Id4 appears to exhibit unique regulatory functions in diverse cancer types. Collectively, these diverse and conflicting results points towards a complex function of Id4 that is probably dependent on a particular genetic background.

A recent report suggested a positive association between Id4 expression and prostate cancer metastasis [77]. In this report, the authors used an anti-Id4 antibody that cross-reacts with multiple proteins in Western blot analysis in our laboratory. On the contrary, our analysis of five independent prostate cancer microarray databases strongly suggests that Id4 is down-regulated in prostate cancer. The development and use of a highly selective antibody will thus ensure the quality and consistency of the data that will significantly advance our understanding of Id4 protein expression in cancers.

## Conclusion

The data strongly supports the role of Id4 as a putative tumor suppressor that may act as a regulatory gene by redirecting cell growth and differentiation. Nevertheless, the opposing functions of Id4 in cancer cells suggest that its molecular mechanism of action may depend on the genotype that may be permissive for its pro- or anti-neoplastic function. This permissive genotype or a "switch" will essentially be determined by the presence/absence of specific Id4 interactions partners or signaling mechanisms that modify response to trophic factors.

It is necessary to analyze Id4 methylation status and expression levels in primary tumor samples as well as in normal prostate tissues in order to further clarify the role of epigenetic silencing of Id4 in prostate cancer. Since epigenetic aberrations are pharmacologically reversible, DNA methylation changes have become attractive targets for cancer treatment with de-methylating agents, leading to reactivation of silenced genes. The identification of novel target genes that may undergo epigenetic silencing in prostate cancer will be important for the development of future therapeutic and diagnostic strategies for clinical application.

## Competing interests

The authors declare that they have no competing interests.

## Authors' contributions

JPWC and AJA contributed equally to the manuscript. AJA developed the DU145-Id4 cell line and JPWC performed subsequent analysis. OG performed methylation analysis of the Id4 promoter. TAG performed androgen receptor western blot and immuno-cytochemistry. JC participated in the study design, reviewed all data and prepared the manuscript. All authors read and approved the final manuscript.

## Pre-publication history

The pre-publication history for this paper can be accessed here:

http://www.biomedcentral.com/1471-2407/9/173/prepub
